# Examining the Effects of Acute Cognitively Engaging Physical Activity on Cognition in Children

**DOI:** 10.3389/fpsyg.2021.653133

**Published:** 2021-05-20

**Authors:** Chloe Bedard, Emily Bremer, Jeffrey D. Graham, Daniele Chirico, John Cairney

**Affiliations:** ^1^Department of Health Research Methods, Evidence, and Impact, McMaster University, Hamilton, ON, Canada; ^2^INfant and Child Health (INCH) Lab, Department of Family Medicine, McMaster University, Hamilton, ON, Canada; ^3^Faculty of Kinesiology and Physical Education, University of Toronto, Toronto, ON, Canada

**Keywords:** affect, exercise, executive function, cognitive engagement, motivation

## Abstract

Cognitively engaging physical activity (PA) has been suggested to have superior effects on cognition compared to PA with low cognitive demands; however, there have been few studies directly comparing these different types of activities. The aim of this study is to compare the cognitive effects of a combined physically and cognitively engaging bout of PA to a physical or cognitive activity alone in children. Children were randomized in pairs to one of three 20-min conditions: (1) a cognitive sedentary activity; (2) a non-cognitively engaging PA; and a (3) cognitively engaging PA. Executive function (EF) was assessed using a modified Eriksen flanker task immediately before and 10–15 min following the experimental condition. Children ages 6–8 years (*n* = 48, Mage = 7.04, *SD* = 1.37; 40% girls) were included in the study. A repeated measures ANOVA found no significant difference between groups with respect to scores on the flanker task. The results do not support the hypotheses that a cognitively engaging bout of PA enhances cognitive performance over non-cognitively engaging PA or sedentary activities. Possible explanations for our findings include overexertion during the acute bout of PA and depletion of positive affect prior to performing the post-intervention EF tasks.

## Introduction

Executive functions (EFs) are the cognitive processes that allow us to mentally hold and manipulate information, ignore or suppress cognitive, visual, verbal, and behavioral stimuli, and shift between tasks and perspectives (Diamond, 2013). Respectively, these EFs are termed working memory, inhibition, and cognitive flexibility, and together they form the foundations for higher-order functions such as planning, reasoning, and problem solving (Diamond, 2013). EFs begin to develop during early childhood and continue developing into adulthood (De Luca et al., 2003). The development of EFs are critical as they contribute to overall development in mental, physical, social, psychological, and academic domains (Diamond, 2013). Deficits in EFs at a young age are associated with poor health, poor economic success, and higher rates of delinquency in adulthood (Moffitt et al., 2011). There is increasing recognition that improvements in EFs in childhood can influence a wide range of developmental domains and have important benefits in adulthood, including better quality of life, increased abilities to obtain and retain a job, and increased likelihood of marital harmony (Diamond, 2013). Furthermore, there are short-term consequences of EFs that affect performance and success in school (Blair and Diamond, 2008). For example, poor EFs tend to associate with lower math and literacy skills in kindergarten (Blair and Razza, 2007) and attention at the age of school-entry is among the three strongest predictors of later academic achievement in elementary school (Duncan et al., 2007).

There is a growing interest in designing both acute and long-term interventions for children and youth that target EFs, such as computer training programs or various types of physical and exercise programs (Diamond and Lee, 2011). The cognitive effects of acute aerobic exercise among children has been studied extensively (Chang et al., 2012; Verburgh et al., 2014). Two systematic reviews reported small to moderate effects of acute physical exercise on several measures of EFs, with the largest gains in inhibition in individuals ages 6–35 years of age (Chang et al., 2012; Verburgh et al., 2014). However, the results across studies vary substantially suggesting that the type and quality of aerobic activity may be important when characterizing this relationship. Chang and colleagues (Bandura, 1997; Chang et al., 2012) noted that duration and intensity of physical activity (PA) moderate this relationship such that activities of at least 11 min of moderate to vigorous intensity produces positive cognitive benefits following a slight delay after the PA bout. However, these systematic reviews were able to identify only a few studies examining these effects in young children (children <12 years of age). At present therefore, it is unclear if these findings are relevant to preadolescent children.

Emerging research has suggested that physical activities that require cognitive engagement are likely to produce superior cognitive benefits compared to non-cognitively engaging PA (Best, 2010; Diamond and Lee, 2011). One potential mechanism for this is that cognitively engaging PA directly recruits the same frontal-dependent neural networks used when EFs are activated. Increased activation of these neural networks during a bout of PA may lead to more efficient neural functioning during cognitive tasks that follow this exercise resulting in enhanced performance (Best, 2010; Diamond and Ling, 2016). Alternatively, or in addition, combining cognitive and physical activities may produce synergistic effects due to co-activation and inter-connectedness of the neural areas associated with cognition and movement (referring broadly to the prefrontal cortex and the cerebellum, respectively). This neural co-activation is strongest when the task is demanding, novel, requires concentration, and when the required response is unpredictable and quick (Diamond, 2000). Therefore, cognitively engaging physical activities may stimulate the necessary contextual parameters to elicit co-activation resulting in enhanced cognitive performance.

Research to date has demonstrated inconsistent findings with respect to this hypothesis, with some studies showing a positive effect of cognitively engaging PA (Budde et al., 2008; Schmidt et al., 2015), and others showing no benefit (Best, 2012; Van den Berg et al., 2016) or even negative effects (Egger et al., 2018). These contradictory findings may be due to differences in intensity of either the cognitive or physical demands of the task (e.g., exergaming, gross motor stations, running), and/or the specific type of EF assessed (e.g., concentration, attentional inhibition). Variability in design and measurement renders comparisons across existing studies problematic.

Beyond design of the task and measurement issues related to EFs, there are other characteristics which may explain or enhance the promotive effect of cognitively engaging PA on EFs, including mood state or affect prior to or during a cognitive task, and personal-motivational factors such as perceived difficulty of the cognitive task, the level of motivation to perform a task, and self-efficacy to perform cognitive tasks. EFs are enhanced when an individual has a feeling of positive affect rather than negative feelings. There is substantial evidence from cognitive psychology that demonstrates improved creativity and cognitive flexibility in participants when a positive mood is induced (Murray et al., 1990; Hirt et al., 2008). Conversely, a depressed mood can impair the ability to focus attention (Desseilles et al., 2009). Given the relationship between mood and cognition, it follows that cognitively engaging PA that induces a positive mood may produce larger benefits to EFs (Diamond and Ling, 2016). While there is very limited evidence that directly supports this hypothesis, Schmidt et al. (2016) demonstrated that increases in positive affect led to better and quicker performance on tests of attention (Schmidt et al., 2016). Therefore, it is important to build in aspects of fun and joy and measure participants’ affective states during these cognitively engaging physical activities. Lastly, the extent of internal motivation, perceptions of task difficulty, and beliefs of self-efficacy are relevant personal-motivational factors that are thought to influence cognitive performance (Puustinen and Pulkkinen, 2001). According to metacognition models, personal-motivational states are highly influential during the performance of cognitive tasks (Borkowski et al., 2000). Generally, children attribute their success or failures on cognitive tasks to factors such as their own ability, effort, attitude, task difficulty, assistance from others, physical factors, or luck. Depending on whether attributions have an internal or external locus, are stable or variable, are controllable or uncontrollable, children may display or feel specific psychological consequences. For example, children may have high feelings of self-efficacy if their attributions of success are internal (i.e., their effort), have expectations of success if attributions of success are stable (i.e., their ability), or have feelings of anger if attributions of failure are controllable (i.e., task difficulty). These affective reactions can affect future cognitive performance as they influence their persistence and willingness to try challenging tasks (Bandura, 1997; Borkowski et al., 2000). Therefore, assessment of motivation to complete cognitive tasks, perceptions of task difficulty, and feelings of task-related self-efficacy are all relevant variables to monitor in studies of interventions to improve cognition, however currently, there is no research relating acute bouts of PA to these personal-motivational factors among children.

These gaps are especially noteworthy in young children, who as we previously noted, are under-represented in studies of EFs and PA in general. EFs develop rapidly between the ages of 5–8 years, with slower gains thereafter (Best et al., 2011; Diamond, 2013). Schmidt et al. (2015) suggests that developing EFs should be more sensitive to changes compared to EFs that are more fully developed in older children. Therefore, younger children under the age of 8 years may be more responsive to PA intervention.

Given the inter-study variations in effects of cognitively engaging PA previously mentioned, and the gap in literature investigating the influence of affective and personal-motivational factors, the objectives of the current study are as follows:

1. Primarily, to evaluate the effect of cognitively engaging PA compared to non-cognitively engaging PA, and cognitively engaging sedentary activity on an EF (inhibition) among 6–8-year-old children. We hypothesized that cognitively engaging PA would show larger effects on inhibition when compared to non-cognitively engaging PA and cognitively engaging sedentary activity.
2. Secondarily, to evaluate the effect of cognitively engaging PA compared to non-cognitively engaging PA, and cognitively engaging sedentary activity on motivation to perform an EF task, perceptions of an EF task difficulty, and beliefs of self-efficacy to perform an EF task. Given the relative novelty of these measures within this research paradigm, we did not have specific hypotheses regarding this secondary objective.

## Materials and Methods

### Design

Three experimental conditions were compared using a randomized controlled trial. Children were randomized in pairs to one of three 20-min experimental conditions that varied in their cognitive and physical demands. Simple randomization with a 1:1:1 ratio was completed using a computer algorithm after eligibility had been established and the participant was enrolled. All assessments were standardized in content and order across the groups. An EF task was administered to all children before and after the experimental manipulation to evaluate within-participant change.

### Participants

A convenience sample of 50 participants was recruited from local youth clubs, community centres, and community events in Southwestern Ontario, Canada. Participants were eligible if they were between the ages of 6–8 years, 12 months and did not have any diagnosed developmental delays or conditions that would prohibit participation in PA. Two children did not complete the full experimental protocol and were therefore excluded from the analysis. Therefore, the final sample consisted of 48 children (mean age = 7.04, *SD* = 1.37; 40% girls). Informed written consent was obtained from all parents/guardians of the participants and children 7 years and older provided written informed assent. Ethical approval for the study was obtained from Hamilton Health Sciences at McMaster University.

The sample size is based on a previous study (Palmer et al., 2013) examining the effect of acute physical activity on sustained attention (one facet of EF), which produced an effect size of *f* = 0.8. Using a conservative estimate with a different intervention, we scaled down the effect to *f* = 0.4. The primary hypothesis relates to a repeated measures analysis of variance (ANOVA) for which a sample size calculation was based, assuming 80% power and a two-tailed alpha of 0.05. This calculation indicated a total sample of 48 (16 in each group) would be sufficient for the analysis (G^∗^Power3.1) (Faul et al., 2009).

### Procedures

Following enrollment, participants were scheduled to visit the lab with another participant. Participants were unaware of their random assignment until they arrived for their study appointment. After consent and assent were obtained, both participants were introduced to each other, and anthropometric assessments were completed. Standing height was measured without shoes to the nearest 0.1 cm using a calibrated stadiometer, and body mass was measured without shoes and the child wearing light clothing to the nearest 0.1 kilogram (kg). Children were then fitted with a Polar heart rate monitor and familiarized with the scales assessing perceived physical and mental exertion and feeling state. Children were then asked to sit quietly without moving for 10 min while monitoring their heart rate variability (this data will not be presented); during this time, children were shown a nature video to maintain attention without being over-stimulating. The children were asked to complete a brief questionnaire measuring their motivation to complete the following cognitive tasks, which took approximately 2 min to complete. The EF task was then administered; participants were each seated at a desk on opposite sides of an auditorium (approximately 20 m apart) to minimize distractions. The EF task lasted approximately 15 min, followed by 20 min of their randomized experimental condition. Immediately after the experimental condition, children were once again seated at their respective ends of the auditorium and asked to again sit quietly without moving for 10 min while viewing the same nature video to reassess their heart rate variability (this data will not be presented). Then children were asked to complete the same questionnaire about motivation in addition to a task self-efficacy questionnaire; this took approximately 3 min. The post-test EF task was then administered, concluding the appointment. While the participant was completing the experiment, the parent (or guardian) was asked to complete a demographic questionnaire and the Behavior Rating Inventory for Executive Functions (BRIEF).

### Experimental Conditions

Each condition lasted 20 min. The cognitively engaging sedentary activity condition (Cognitive Group; *n* = 16) consisted of two children seated at opposite sides of a table playing the game Connect 4 (board dimensions: length 26 cm; height 20 cm). Once a game was won, the board would be re-set and the children would start a new game. The non-cognitively engaging PA condition (Exercise Group; *n* = 16) consisted of children running to and from a pylon placed 45 feet in front of them, alternating turns. The cognitively engaging PA condition (Dual Group; *n* = 16) consisted of children playing a large game of Connect 4 (board dimensions: length 121 cm; height 117 cm) which was placed 45 feet in front of them; children were asked to run to the board to play the game. Once a game was won, the board was re-set and a new game would begin.

### Measures

#### Background Variables

The demographic questionnaire included questions about the parent and the child on age, gender, race/ethnicity, parental education and occupation, and household income. Parents also completed the Behaviour Rating Inventory of Executive Functions (BRIEF) (Gioia et al., 2001) to assess trait levels of EFs. This assessment is appropriate for parents of children ages 5–18 years and takes approximately 10–15 min to complete. The instrument assesses the following domains of EF: inhibition, shifting, emotional control, working memory, and planning/organizing. Scores are totaled to give the Global Executive Composite. Parent test-retest reliability scores from the normative sample of 1,419 parents of children aged 5–18 is 0.82. Anthropometric measurements of body mass and height were used to compute body mass index (BMI; kg/m^2^) and International Obesity Task Force (IOTF) guidelines were used to determine the thresholds for thinness, overweight and obesity by age and sex (Cole and Lobstein, 2012).

#### Manipulation Check Variables

Physical exertion was measured both objectively using heartrate (HR) data collected during the experimental condition and subjectively using the 12-point Borg RPE scale (Borg, 1982). HR was recorded every 60 s and perceived physical exertion was assessed every 4 min throughout the 20-min conditions. HR in the age predicted zones of moderate to vigorous intensity (approximately 135–160 bpm) were intended to create conditions most likely to produce the largest gains in cognition (Chang et al., 2012). Cognitive exertion was also measured subjectively every 4 min during the 20-min conditions using a modified Borg RPE scale which asks participants to provide their rating of perceived mental exertion (RPME) on the same RPE scale ranging from 1 to 12. Affect was assessed using the Feeling Scale (FS) (Hardy and Rejeski, 1989) which asks participants to rate their current affective (or feeling) state using a bipolar scale ranging from -5 (feeling very bad) to + 5 (feeling very good). Ratings of RPE, RPME, and FS took approximately 10–20 s to complete.

#### EF Task

A modified Eriksen Flanker task was used to assess attentional inhibition (Eriksen and Eriksen, 1974). The task was administered on an iPad (iPad Mini 2) and took approximately 15 min to complete. Children were presented with an image of five fish on a horizontal plane and the task required children to tap an arrow on either side of the screen indicating the direction of the middle fish. The four fish flanking the middle fish were either facing the same direction as the middle fish (congruent) or the opposite direction of the middle fish (incongruent). The image was presented for 200 ms and participants were asked to respond as quickly as possible by tapping their right or left index finger (depending on the direction of their response) on the corresponding arrow on the screen. A response window was limited to 2,000 ms before presenting the next image. After making a response, children returned their index finger to “home base” which were two large black dots (one for each finger) taped on the desk, 4 cm from the base of the iPad. Ten practice trials presented at 2,000 ms and 20 practice trials presented at 200 ms were administered with appropriate feedback, then the children completed 4 blocks of 50 trials with no feedback; participants rested for 2-min between blocks (Chang et al., 2013). Each block contained a random sequence of congruent right, congruent left, incongruent right, incongruent left with 40% of trials congruent and 60% incongruent (maximum number of repeated trials was limited to five). Trials in which the reaction time was <150 ms or >3 SD from the child’s mean were excluded from analysis. Interference effects (incongruent–congruent) were calculated for inverse efficiency, response time (RT), and accuracy and were used as the primary dependent variables. Inverse efficiency measures overall performance by combining both RT and accuracy rates in the following calculation: mean RT on correct trials/proportion correct.

#### Personal-Motivational Variables

Motivation for performing the flanker task was assessed using the 5 items from the effort and importance subscale from the Intrinsic Motivation Inventory (IMI) which took approximately 2 min to complete (Ryan, 1982). The IMI was administered prior to completing the flanker task before and after the experimental conditions. To assess perceived task difficulty for performing the flanker task, children were asked to rate their RPME within the 2-min rest periods; average ratings were computed for the pre- and post-experimental manipulation administrations of the flanker task. Task Self Efficacy for completing the flanker task following the experimental manipulation was assessed using a 4-item scale adhering to recommendations from Bandura for assessing self-efficacy (Bandura, 1997, 2006). Each item reflected degrees of performance relative to the participant’s performance on the first executive function task. The first item asked participants to rate their confidence that they would perform the executive function task “almost as good as last time,” followed by “as good as last time,” then “a little bit better than last time, “and finally “A lot better than last time.” The participants rated their confidence on an 11-point Likert scale. The average score across these 4 items comprised the total self-efficacy score.

### Statistical Analysis

All data analyses were performed using SPSS version 25 (SPSS, 2010). Descriptive statistics were used to describe the participants (see Table 1). To test for imbalances across groups on demographic factors and baseline EF and motivation one-way ANOVAs were conducted with group as the independent variable.

**TABLE 1:** Demographic and baseline characteristics by condition.

**Variable**	**Cognitive group**	**Exercise group**	**Dual group**	***p***
Age, mean (SD)	7.33 (1.35)	7.04 (0.54)	6.74 (1.86)	0.48
Female; *n* (%)	5 (31.25)	8 (50.00)	6 (37.50)	0.56
BMI IOTF				0.21
Thinness; *n* (%)	0 (0)	1 (6.25)	0 (0)	
Normal weight; *n* (%)	15 (93.75)	14 (87.50)	13 (81.25)	
Overweight; *n* (%)	1 (6.25)	0 (0)	2 (12.50)	
Obese; *n* (%)	0 (0)	1 (6.25)	1 (6.25)	
Child ethnicity*				0.73
Latin American	0 (0)	1 (6.67)	0 (0)	
White	15 (93.75)	13 (86.67)	13 (86.67)	
Mixed race	0 (0)	1 (6.67)	0 (0)	
South Asian	1 (6.25)	0 (0)	1 (6.67)	
Southeast Asian	0 (0)	0 (0)	1 (6.67)	
Caregiver age	39.87 (5.04)	38.20 (4.41)	40.50 (7.33)	0.54
Global executive composite Score	46.13 (9.87)	49.13 (7.86)	49.27 (6.53)	0.49
Baseline motivation	5.80 (1.09)	5.61 (0.96)	5.94 (1.11)	0.68
Baseline executive function				
Interference accuracy	−0.10 (0.20)	−0.11 (0.13)	−0.07 (0.06)	0.78
Interference response time	71.79 (70.09)	56.72 (65.82)	96.60 (37.80)	0.17
Interference inverse efficiency	526.30 (1397.79)	336.58 (475.52)	206.80 (125.10)	0.57

*Exercise and dual groups missing 1 for ethnicity; SD, standard deviation; n, sample size; BMI, body mass index; IOTF, International Obesity Task Force.

To check assumptions of the manipulation, four separate one-way ANOVAs were run for the average RPME, RPE, HR, and FS measured during the experimental conditions.

The primary analyses were three repeated measures ANOVAs with group as the independent variable and the interference effects for the inverse efficiency score, response time, and accuracy as dependent variables, respectively. Only analyses that met statistical assumptions of repeated measures ANOVA are presented; if assumptions were violated the analysis was re-run using a dataset excluding participants in whom their change score on the dependent variable exceeded three standard deviations of their group mean. The statistical assumptions of homogeneity of the intercorrelations could not be met in the analysis examining the interference accuracy score with either a full dataset or one with removed outliers, therefore it will not be presented.

Secondary analyses were repeated measures ANOVA with group as the independent variable and personal-motivational variables as the dependent variable: intrinsic motivation and average RPME during the EF task. Since task-self efficacy was only measured once prior to the second EF task an ANOVA was completed to test the effect of group on this variable.

All significance thresholds were set to *p* < 0.05 and partial eta squared was used to describe the magnitude of the effects. Multiple comparisons were not adjusted for, therefore inferences drawn from the results of the secondary and exploratory analyses might not be fully reproducible.

## Results

There were no significant differences between groups on parental age or income, participant age, sex, ethnicity, global executive composite, BMI, baseline motivation, or EF scores (see Table 1).

### Manipulation Checks

**FIGURE 1:**
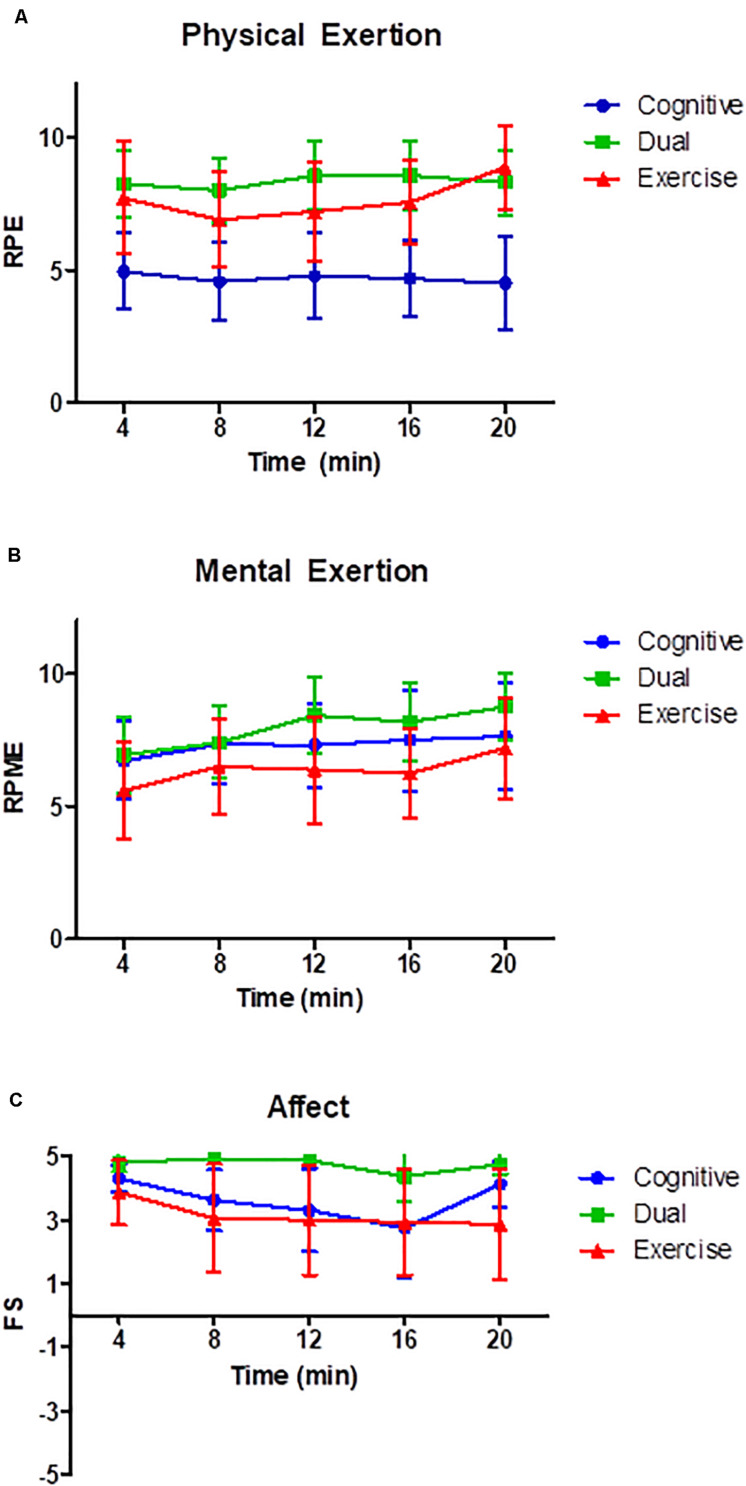
**(A–C)** Ratings of physical exertion **(A)**, mental exertion **(B)**, and affect **(C)** during the experimental manipulation by condition. RPE, ratings of perceived physical exertion; RPME, ratings of perceived mental exertion; FS, feeling state.

Significant differences were found for RPE, HR, and FS (see Figures 1A–C and Table 2) between the experimental groups. Significantly higher RPE was present in the two PA groups [*F*_(2, 45)_ = 10.98, *p* < 0.001, partial η*^2^* = 0.33] compared with the cognitive group (no significant difference between the exercise and dual groups, *p* = 0.41). Similarly, the average HR was significantly higher in the two PA groups [*F*_(2, 45)_ = 124.24, *p* < 0.001, partial η*^2^* = 0.85] compared to the cognitive group (no significant difference between the exercise and dual groups, *p* = 0.39). Children in both PA groups performed at an average of 75% of their maximal heart rate using the formula, 220-age (Fox et al., 1971) which corresponds to a moderate to vigorous intensity. There was an overall significant difference in feeling state [*F*_(2, 45)_ = 3.50, *p* < 0.05, partial η^2^ = 0.13] with children in the dual group reporting significantly higher positive feelings compared to the exercise group (no significant differences between dual and cognitive groups, *p* = 0.08). Children in the cognitive and dual conditions reported higher levels of mental exertion when compared to the exercise group, however, this was not significant (*p* = 0.30). The results suggest that the experimental manipulations were successful in producing the expected difference in physical exertion and enjoyment. Despite non-significant differences in cognitive exertion, the pattern of group means demonstrate that the cognitive and dual groups elicited higher levels of perceived cognitive demands compared to the exercise group.

**TABLE 2:** Manipulation checks by condition.

**Variable**	**Cognitive group**	**Exercise group**	**Dual group**	***p*-value**
HR	88.37 (9.76)	164.66 (22.11)*	159.92 (11.04)*	<0.001
RPE	4.69 (2.46)	7.65 (2.55)*	8.34 (1.96)*	<0.001
RPME	7.30 (2.90)	6.38 (3.11)	7.94 (2.34)	0.29
FS	3.62 (1.48)^†^	3.15 (2.65)	4.75 (0.43)^†^	0.04

HR, heartrate; RPE, ratings of perceived physical exertion; RPME, ratings of perceived mental exertion; FS, feeling state; *significantly different from the cognitive group; ^†^significantly different from the exercise group.

### Primary Analyses

There were no significant differences between groups on either interference inverse efficiency scores or interference response time (see Table 3), however, the direction of effect favored the cognitive group over the PA groups.

**TABLE 3:** Primary and secondary analyses: repeated measures ANOVA effect of time by group.

**Variable**	***F***	***P***	**Partial eta square**
Interference RT	0.25	0.78	0.01
Interference inverse efficiency	1.26	0.30	0.06
Intrinsic motivation inventory	0.36	0.70	0.02
Average ratings of perceived mental exertion during flanker	0.11	0.90	0.01
Task self-efficacy for completing second Flanker*	0.28	0.76	0.01

*One-way ANOVA; RT, response time.

### Secondary Analyses

Secondary analyses showed no significant differences between groups for any of the personal-motivational variables (see Table 3).

## Discussion

The primary objective of this study was to evaluate the effect of various cognitively and physically engaging activities on an EF in 6–8-year-old children. It was hypothesized that cognitively engaging PA would have superior effects on cognition. Overall, however, the results do not support our hypothesis: we found no significant differences in any of the EF metrics between the dual, exercise, or cognitive group. Surprisingly the cognitively engaging PA group appeared to perform worse, compared to the other two groups, and there was no advantage of the exercise group over the cognitive group. Furthermore, the results also showed non-significant differences in the effect of the activities on the personal-motivational factors. In general these results are misaligned with other research in the field which supports the beneficial cognitive effect of acute PA on cognition (Budde et al., 2008; Pesce et al., 2009; Benzing et al., 2016), however our results are consistent with the few that show no or detrimental cognitive effects (Best, 2012; Egger et al., 2018). Neither PA group produced improvements in their scores on the EF task of inhibition, despite following the prescribed activity duration and intensity of 20 min of moderate to vigorous activity likely to produce cognitive benefits (Chang et al., 2012). Task constraints and procedural limitations may be responsible for the inconsistency between our results and previous research.

### Task Constraints

Recommendations from the meta-analysis conducted by Chang et al. (2012), suggest that 20 min of moderate to vigorous physical activity (MVPA) will produce cognitive improvements. However, a duration of 20 min may have been too long for this young sample of 6–8-year-old children. The evidence from which the recommendation of 11–20 min of MVPA are based upon are mainly derived from samples of young adults, with fewer studies conducted with younger samples. Therefore, the dose-response relationship between activity duration and cognitive effects may need to be shifted to shorter durations of activity. This hypothesis is supported by the results of Howie et al. (2014) which found significant improvement in academic outcomes in a classroom-based PA session of 15 min, and no improvement after 20 min. Egger et al. (2018) also argues for a shorter duration of activity as their 20-min classroom-based cognitively engaging PA session also showed no significant effect on inhibition and actually led to worse performance on a shifting task.

An additional task constraint which may have affected the results is the intensity of the PA. Chang et al. (2012) found that when the cognitive task is performed following a delay after exercise, higher intensities produced the largest effect on cognition. However, this finding once again may not be applicable to a younger sample. In the current study, children in both PA groups were performing at an estimated 75% of their age-predicated max heartrate; while still in a moderate-to-vigorous intensity range, this may have overexerted the young sample. Budde et al. (2008) reported an average HR of 120 bpm in a sample of 13–16 year old participants and demonstrated positive effects of their cognitively engaging PA session on cognition. Best (2012), however, had participants performing between 70 and 80% of their max HR and they were also not able to produce superior cognitive effects following cognitively engaging exergaming compared to non-cognitively engaging exergaming among 6–10 year old children. Therefore, it is plausible that PA of lower intensity may be more likely to elicit cognitive benefits than more vigorous activities among younger children. This is consistent with the strength model of self-control which posits that individuals have a finite reserve of mental resources to draw upon when performing cognitive tasks. This reserve can be spent when we engage in activities that require self control or activation of EFs, such as intense exercise (Baumeister et al., 1998; Audiffren and André, 2015). Children participating in both PA groups reported high levels of both physical and mental exertion, therefore suggesting that high levels of self-control were required to continue to engage in the 20-min activity. It is possible that the flanker task performance was impaired by overexertion during both PA conditions, therefore the participants’ mental capacity was drained and consequently they could not perform the task efficiently. Although not significant, the cognitive group appeared to have a better performance on the flanker task following their activity, possibly because they had not depleted their cognitive resources during the seated cognitive game and could therefore utilize their cognitive reserve.

### Procedural Constraints

Beyond the activity itself, the non-significant findings of this study may be explained through procedural limitations, specifically with respect to the choice of EF task, timing of EF task administration, and activities that preceded the EF task. In general, training interventions that produce the largest cognitive effects are those that specifically train the EF to be tested; in other words, EF transfer is narrow (Diamond and Ling, 2016). Improvements in inhibition are most likely found after completing an activity that directly trains inhibition. Therefore, it is possible that the assessment of inhibition used in the current study may not have been sufficiently specific to the EF training that the dual activity elicited. The goals of the game are to connect four of your pieces in a row before your opponent. It is critical to be able to visualize your opponents’ options to block their opportunity to connect four; while this certainly demands inhibition to focus on the task, ignore distractions in the environment, and inhibit the impulse to follow a planned strategy when the opponent can connect four pieces in their next turn, it also requires a high degree of cognitive flexibility, to be able to shift perspectives and change priorities (i.e., from winning to blocking). Therefore, Connect 4 may train cognitive flexibility in addition to inhibition, so the flanker task of inhibition may not have been sensitive or responsive to any enhanced training effects. Future research should consider the specific training that is occurring throughout the intervention and accordingly administer the relevant EF task to assess training effects. Alternatively, multiple facets of EFs could be assessed following an intervention to evaluate the extent of transfer of EF training during cognitively engaging PA.

An additional consideration of the current study is the timing of the EF testing, such that it followed a 13-min delay after the activity. The meta-analysis from Chang et al. (2012) showed the largest effects when the cognitive task was administered between 1 and 15 min. Therefore, administration following a 13-min delay may have been too long to detect any positive gains in the EF possibly elicited from the activity. However, given the divergent results from Chang et al. (2012) with respect to duration and intensity among young children it is unclear if the same effects of immediate testing would hold true in a younger sample. For example, Egger et al. (2018) administered EF tasks immediately following the intervention sessions and did not find a positive benefit to cognition among a sample of 7–9-year-old children. More research is necessary among younger children to ascertain relationships between timing of EF task administration following a bout of PA.

**FIGURE 2:**
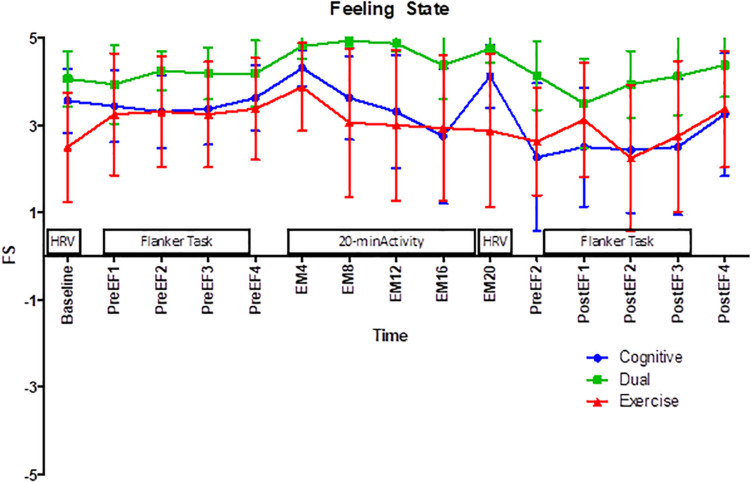
Repeated assessment of feeling state across the study protocol. EF, executive function; EM, experimental manipulation; HRV, heart rate variability assessment.

Finally, an important influencing factor involves consideration of the events that immediately preceded the second test of inhibition in the current study. Immediately after the experimental manipulation was complete, the children were asked to sit still to return their heartrate to resting state and reassess heartrate variability; during this 10-min break they viewed the same nature video. Repeated assessment of feeling state revealed that across all groups there was a significant decrease in positive affect during this 10-min break (see Figure 2). Therefore, gains in affect produced by the 20-min dual activity were depleted prior to completing the second test of EF, possibly contributing to lowered performance on the flanker task. The “mood as facilitator” theory states that a positive feeling state stimulates other positive reflections and thoughts, which results in more efficient EFs (Dalgleish and Power, 2000; Isen and Reeve, 2005). In a study of the cognitive effect of classroom-based PA, Schmidt et al. (2016) found support in the “mood as a facilitator” theory such that change in positive affect during the intervention led to improved scores of attention and processing speed. Given this relationship between positive affect and cognitive performance it is possible that if the positive affect levels of the cognitive engaging PA group had been maintained, cognitive gains may have been observed. However, the reduction of positive affect following the activity may have disabled mood as a potential facilitator of an enhanced performance on the EF task.

### Absence of an Effect on Personal-Motivational Factors

Given the relative novelty of including measures of task self-efficacy, perceptions of task difficulty, and motivation to perform the EF task in trials investigating the effects of cognitively engaging PA we did not enter into the secondary objective of the study with any specific hypotheses. Nonetheless, given the non-significant group differences in inhibition and the reduction in positive affect following the 20-min intervention activities (alongside previous research from Schmidt et al., 2016), it is not surprising that the results also show no differences between groups in any of the measured personal-motivational factors. It is plausible that the reduction in positive affect influenced the personal-motivational factors in a similar manner. Perhaps if the positive gains in affect were maintained throughout the 13-min delay after the activity, the personal-motivational status would have been enhanced, and this may have subsequently improved the EF performance. Indeed, positive affect is associated with higher perceptions of self-efficacy and, in turn, performance (Bandura, 1997). Alternatively, Schmidt et al. (2016) suggests that positive affect related to high motivational intensity, that is positive affect which results in the motivation to improve performance, may be more relevant than generalized positive affect associated with low motivational intensity. It is possible that the positive affect gained throughout the cognitively engaging PA in the current study was related to a low motivational intensity and thus changes in motivation and inhibition were not observed. Given the drastic drop in positive affect following the intervention, it is unknown how specifically motivation and affect were related. Despite non-significant findings, personal-motivational variables are still relevant factors that should continue to be considered and measured.

### Limitations and Future Directions

Beyond the aforementioned limitations, there are several others in this study which should be identified. First, the validity and reliability of the measurements of perceived physical and mental exertion and feeling state among young children is unknown. To ensure adequate comprehension of the scales, the children received a thorough explanation of each of the exertion and feeling state scales prior to the start of the protocol. Additionally, each of these scales have been used successfully in other studies within this research team in samples of children of similar age. The results of the manipulation checks provide some confidence in their validity, however, their sensitivity to small changes and reliability is still undetermined. Additionally, the assessment of internal motivation and self-efficacy are also not formally validated in samples of young children, therefore inferences based on these assessments should be cautioned and replicated. These measurement limitations reflect generalized shortcomings of this field since research still lacks an appropriate measure of these constructs in young children. Also, since the sample size calculation was designed from a study with a younger sample and different outcome instrument, it is likely that the analysis was underpowered with only 16 participants in each group. Finally, this study did not include a control group of neither physical or cognitive activity, and therefore cannot be directly compared to studies examining the impact of either PA or cognitively engaging PA on cognition compared to no activity. However, given the scope of the research question and in consideration of the context of results, the addition of this type of control would have yielded negligible value.

## Conclusion

Overall, the results of this study contradict our hypotheses and suggest that a bout of cognitively engaging PA does not produce superior effects on cognition, however, this may be a result of overexertion during PA and a reduction in positive affect following the experimental manipulation. Future research should be directed toward investigating the optimal physical and cognitive load during activities to produce cognitive benefits, and the role of affect and personal-motivational factors in this relationship.

## Data Availability Statement

The datasets presented in this article are not readily available because due to the nature of this research, participants of this study did not agree for their data to be shared publicly, so supporting data is not available. Requests to access the datasets should be directed to CB, c3bedard@uwaterloo.ca.

## Ethics Statement

The studies involving human participants were reviewed and approved by the Hamilton Integrated Research Ethics Board at McMaster University. Written informed consent to participate in this study was provided by the participants’ legal guardian/next of kin.

## Author Contributions

CB designed the study, coordinated recruitment and data collection, conducted data collection, carried out the data analyses, and was the primary author of the manuscript. EB, JG, and DC advised on study design, assisted with data collection, and revised and approved the final manuscript as submitted. JC supervised the design and execution of all phases of the study and revised and approved the final manuscript as submitted. All authors contributed to the article and approved the submitted version.

## Conflict of Interest

The authors declare that the research was conducted in the absence of any commercial or financial relationships that could be construed as a potential conflict of interest.
